# Protecting the heart

**DOI:** 10.7554/eLife.91831

**Published:** 2023-09-06

**Authors:** Joshua M Inglis, Arduino A Mangoni

**Affiliations:** 1 Department of Clinical Pharmacology, Flinders Medical Centre and Flinders University Adelaide Australia; 2 Adelaide Medical School, University of Adelaide Adelaide Australia

**Keywords:** doxorubicin, cardiotoxicity, EPAC1, cancer survivorship, Human, Mouse, Rat

## Abstract

Blocking a protein known as EPAC1 may prevent the development of heart-related side effects caused by a chemotherapy drug.

**Related research article** Mazevet M, Belhadef A, Ribeiro M, Dayde D, Llach A, Laudette M, Belleville T, Mateo P, Gressette M, Lefebvre F, Chen J, Bachelot-Loza C, Rucker-Martin C, Lezoualch F, Crozatier B, Benitah J-P, Vozenin M-C, Fischmeister R, Gomez A-M, Lemaire C, Morel E. 2023. EPAC1 inhibition protects the heart from doxorubicin-induced toxicity. *eLife* **12**:e83831. doi: 10.7554/eLife.83831.

Advances in medical sciences mean that some cancers can now be cured or treated as chronic diseases which can be managed over long periods of time. This has led to the emerging field of cancer survivorship, which includes preventing and managing the side effects of cancer treatments (Emery et al., 2022).

For example, chemotherapy drugs called anthracyclines are used to treat haematologic cancers and cancers affecting solid organs in both adults and children. However, they can also lead to heart damage in around 20% of adults within two to three years of treatment, and in children up to 30 years after the initial exposure (Limat et al., 2003; Oeffinger et al., 2006; Romond et al., 2012).

Anthracyclines may cause DNA damage that leads to cell death, but it is unclear why the heart is affected over other organs (Tan et al., 2015). Clinicians sometimes use cardioprotective drugs, such as dexrazoxane, to prevent the toxic side effects of anthracyclines (US Food and Drug Administration, 2014). However, cardioprotective drugs are not always used due to concerns that they may reduce how the tumour responds to treatment, as well as causing side effects (Swain et al., 1997; Shaikh et al., 2016). A better understanding of the mechanisms underlying anthracycline-induced cardiotoxicity is therefore essential to develop more effective preventive strategies.

Now, in eLife, Eric Morel, Christophe Lemaire and colleagues – including Marianne Mazevet as first author – report on how blocking a specific protein appears to prevent anthracycline-induced heart damage (Mazevet et al., 2023). The researchers (who are based at various institutes in France, Switzerland and the United States) exposed rat cardiac cells and live mice to an anthracycline called doxorubicin; they then examined how the cardiotoxicity of this drug was affected by the protein EPAC1, which contributes to heart failure by modifying cardiac cells (Surinkaew et al., 2019).

First, Mazevet et al. showed that rat cardiac cells treated with doxorubicin displayed signs of DNA damage, and that signalling enzymes linked to apoptosis were being activated. The drug also increased the activity of EPAC1, as well as its expression. This latter effect was also observed in live mice treated with doxorubicin. However, EPAC2 – the isoform of EPAC1 – does not appear to be involved in the response.

Pharmacological inhibition of EPAC1 in rat cardiac cells reduced the activation of apoptotic pathways caused by doxorubicin; notably, the inhibitor also provided the same level of protection against cardiac toxicity as the cardioprotective drug dexrazoxane. Experiments on live mice provided further insights into the interactions between EPAC1 and doxorubicin. Knock-out animals lacking the EPAC1 gene showed no signs of cardiotoxicity when administered doxorubicin, while wild-type animals displayed increased EPAC1 expression and decreased cardiac contractility after 6 weeks of treatment (Figure 1). However, this knock-out study did not investigate other potential manifestations of cardiotoxicity such as irregular heartbeats or more subtle cardiac abnormalities.

**Figure 1.**
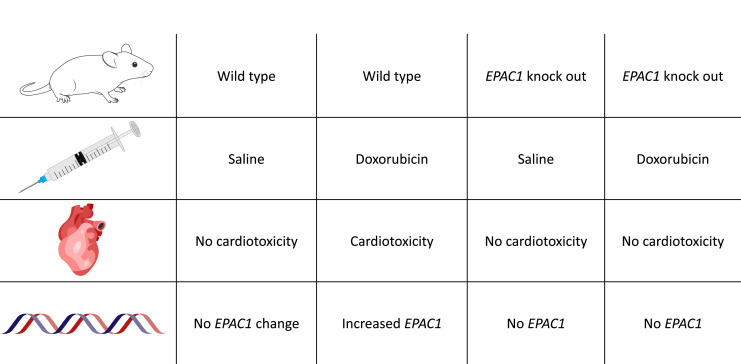
Loss of EPAC1 reduces heart damage in mice receiving doxorubicin. Table showing the prevention of doxorubicin-induced cardiomyopathy in wild-type and *EPAC1* knockout mice (i.e., mice without the *EPAC1* gene). Increased *EPAC1* expression was seen in wild-type mice receiving doxorubicin who also developed cardiomyopathy.

Image credit: Illustrations adapted from SciDraw (CC BY 4.0): mouse by Ethan Tyler and Lex Kravitz, syringe by Hassan Ghayas, heart by John Chilton and DNA strands by Guilherme Bauer Negrini.

Finally, EPAC1 inhibition enhanced the cytotoxic effect doxorubicin had on breast and cervical human cancer cells grown in the laboratory. It will be necessary to confirm the anticancer effects of this drug in live animals, and in combination with the other available anthracyclines. If such combinations were confirmed to have an additive anticancer effect, a lower dose of anthracyclines may then be required, which would further reduce the risk of cardiotoxicity.

In conclusion, Mazevet et al. have demonstrated the critical role of EPAC1 in triggering the signalling pathways involved in anthracycline-induced cardiotoxicity. Both the pharmacologic inhibition of EPAC1 and knocking out the gene for this protein in mice prevented the cancer drug doxorubicin from causing heart damage, suggesting a promising avenue of research for preventing cardiotoxicity in patients treated with anthracyclines.

While pharmacologically inhibiting EPAC1 was shown to be effective in rat heart cells grown in the laboratory, Mazevet et al. did not test the compound in vivo. Also, as only a single concentration of the inhibitor was studied, the potential dose-response relationship remains unclear. Further studies are needed to assess the pharmacokinetics and tolerability of the inhibitor for EPAC1 in animal models, as well as any possible drug interactions. An examination of the molecular structure of the compound may reveal potentially reactive groups. Consideration will also need to be given to the route of administration in future preclinical and human studies.
